# A phase 2b, randomized, placebo-controlled, double-blind, dose-ranging study of the neurokinin 3 receptor antagonist fezolinetant for vasomotor symptoms associated with menopause

**DOI:** 10.1097/GME.0000000000001510

**Published:** 2020-02-24

**Authors:** Graeme L. Fraser, Samuel Lederman, Arthur Waldbaum, Robin Kroll, Nanette Santoro, Misun Lee, Laurence Skillern, Steven Ramael

**Affiliations:** 1 OGEDA SA, a wholly owned subsidiary of Astellas Pharma SA, Belgium; 2 Altus Research, Lake Worth, FL; 3 Downtown Women's Healthcare, Denver, CO; 4 Seattle Women's: Health, Research, Gynecology, Seattle, WA; 5 University of Colorado School of Medicine, Aurora, CO; 6 Astellas Pharma, Inc., Northbrook, IL; 7 Astellas Pharma Europe Ltd, Chertsey, UK.

**Keywords:** Hot flashes, KNDy neuron, Menopause, Neurokinin B, NK3 receptor antagonist, Vasomotor symptoms

## Abstract

**Objective:**

Menopausal vasomotor symptoms (VMS) may result from altered thermoregulatory control in brain regions innervated by neurokinin 3 receptor-expressing neurons. This phase 2b study evaluated seven dosing regimens of fezolinetant, a selective neurokinin 3 receptor antagonist, as a nonhormone approach for the treatment of VMS.

**Methods:**

Menopausal women aged >40-65 years with moderate/severe VMS (≥50 episodes/wk) were randomized (double-blind) to fezolinetant 15, 30, 60, 90 mg BID or 30, 60, 120 mg QD, or placebo for 12 weeks. Primary outcomes were reduction in moderate/severe VMS frequency and severity ([number of moderate VMS × 2] + [number of severe VMS × 3]/total daily moderate/severe VMS) at weeks 4 and 12. Response (≥50% reduction in moderate/severe VMS frequency) was a key secondary outcome.

**Results:**

Of 352 treated participants, 287 completed the study. Fezolinetant reduced moderate/severe VMS frequency by −1.9 to −3.5/day at week 4 and −1.8 to −2.6/day at week 12 (all *P < *0.05 vs placebo). Mean difference from placebo in VMS severity score was −0.4 to −1 at week 4 (all doses *P < *0.05) and −0.2 to −0.6 at week 12 (*P < *0.05 for 60 and 90 mg BID and 60 mg QD). Response (50% reduction) relative to placebo was achieved by 81.4% to 94.7% versus 58.5% of participants at end of treatment (all doses *P < *0.05). Treatment-emergent adverse events were largely mild/moderate; no serious treatment-related treatment-emergent adverse events occurred.

**Conclusions:**

Fezolinetant is a well-tolerated, effective nonhormone therapy that rapidly reduces moderate/severe menopausal VMS.

***Video Summary*:**http://links.lww.com/MENO/A572; video script available at http://links.lww.com/MENO/A573.

Vasomotor symptoms (VMS), consisting of hot flashes and night sweats, are among the most common and bothersome symptoms of menopause. Up to 80% of women in the United States experience VMS during the menopausal transition,^1,2^ and VMS persist for a median of 7.4 years.^3^ Up to two thirds of women who experience VMS find these symptoms bothersome.^4^ Furthermore, VMS are the menopausal symptoms for which women most often seek treatment.^5^ However, in the United States and most European countries, only about 3% to 10% of perimenopausal and postmenopausal women use menopausal hormone therapy (HT),^6-8^ the most effective available treatment for VMS associated with menopause.^9-12^ A recent meta-analysis of prospective studies including more than 100,000 postmenopausal women who developed breast cancer described an increased relative risk of breast cancer associated with use of HT (estrogen alone or combination estrogen/progestogen) as well as with a longer duration of HT use.^13^ Also, systemic estrogen/progestogen therapy has been associated with an increased risk of stroke and venous thromboembolism and with common adverse events (AEs) including breakthrough bleeding, breast tenderness, nausea, bloating, and mood swings.^11,14^ Despite recognition of the continued role for HT in international clinical practice guidelines, especially for symptomatic women aged <60 years or within 10 years of menopause,^9-12,15^ safety and tolerability concerns have discouraged women suffering from VMS from using HT.^6^

The selective serotonin reuptake inhibitor paroxetine is the only US Food and Drug Administration (FDA)-approved nonhormone pharmacologic therapy for VMS.^16^ Paroxetine is considered less effective than estrogen-based treatment, although there are no direct comparative studies.^16,17^ Also, paroxetine is associated with significant nausea and dizziness, as well as fatigue and, in rare cases, bone fracture and suicidal ideation.^16,18^ Moreover, long-term safety data for paroxetine use in postmenopausal women are lacking. Thus, there remains a considerable need for a safe, highly effective, nonhormone therapy for relief of VMS associated with menopause.

Neurokinin-3 receptor (NK3R) antagonism is a novel, nonhormone approach to treating VMS that directly targets the underlying central mechanism thought to be responsible for this medical condition. A subset of hypothalamic neurons that co-express the neuropeptides kisspeptin, neurokinin B, and dynorphin (KNDy neurons) project from the arcuate nucleus to the preoptic area of the hypothalamus and are believed to play a key role in thermoregulation.^19-21^ These neurons are inhibited by estrogen and stimulated by NK3R activation.^22-26^ With declining estrogen levels in menopause, NK3R-mediated signaling is unopposed and hypertrophy of KNDy neurons is observed with anticipated, commensurate changes in the activity of the brain regions that these neurons innervate.^27-29^ The altered activity of this neural circuit results in the thermoregulatory centre becoming hypersensitive to external cues from peripheral sensors, activating heat dissipation effectors (eg, sweating, vasodilation), and is believed to be the physiologic basis for why many menopausal women experience VMS.^20-22^

Fezolinetant, an oral NK3R antagonist that moderates KNDy neuronal activity,^30,31^ is in clinical development for the treatment of moderate/severe VMS associated with menopause. A previous randomized, double-blind, placebo-controlled, phase 2a clinical trial in 87 postmenopausal women showed that fezolinetant 90 mg BID was highly effective in reducing moderate/severe VMS and well tolerated across 12 weeks of treatment.^32^ Herein, we report a phase 2b study (VESTA) conducted to evaluate the efficacy and safety of various doses and dosing regimens of fezolinetant in the treatment of VMS associated with menopause. The primary objective of this study was to evaluate the effect of these regimens on VMS frequency and severity.

## METHODS

### Study design

This was a randomized, double-blind, placebo-controlled, dose-ranging, parallel group study conducted at 51 sites in the United States from July 19, 2017, through September 19, 2018 (NCT03192176). The screening period (days −35 to −1) included a screening visit and baseline assessments of VMS frequency and severity, which were recorded daily throughout screening by participants using an electronic diary. Eligible participants were randomized on day 1 and received 12 weeks of study treatment; visits to study sites occurred at least once every 4 weeks during the treatment period. Follow-up occurred approximately 3 weeks after the last dose of study drug (week 15). Participants continued to record VMS frequency and severity at least twice daily in the electronic diary from screening through the final follow-up visit.

All participants were dispensed a morning bottle and an evening bottle and assigned to take fezolinetant or placebo twice daily. On day 1 of the treatment period, participants were randomly assigned in equal numbers to one of the following treatment groups: fezolinetant 15, 30, 60, or 90 mg BID, or fezolinetant 30, 60, or 120 mg QD (taken in the morning, accompanied by placebo taken in the evening to maintain the blind), or placebo BID. An independent, consulting biostatistician who was not involved in the study prepared the computer-generated randomization schedule using SAS software (SAS Institute, Cary, NC) before the start of the study, which balanced randomly permuted blocks across treatment groups. Participants were assigned a randomization number from an interactive response technology system upon enrollment. Fezolinetant and placebo capsules were identical in appearance and contained the same excipients. Study participants and all members of the study team directly involved in data management or clinical, medical, or statistical review were blinded to treatment allocations.

The study was approved by institutional review boards at each study site and was conducted in accordance with the Declaration of Helsinki and in compliance with International Council for Harmonisation Good Clinical Practice Guidelines. All participants provided written informed consent before study enrollment.

### Study population

Eligible participants included postmenopausal women aged >40 and ≤65 years, with ≥50 moderate/severe VMS episodes per week based on seven consecutive days of VMS recordings from any point during the 35-day screening period. Natural or surgical menopause was defined as meeting one of the following criteria: (a) spontaneous amenorrhea for ≥12 months, (b) spontaneous amenorrhea for ≥6 months with biochemical confirmation of menopause (ie, follicle-stimulating hormone [FSH] > 40 IU/L), or (c) bilateral oophorectomy (with or without hysterectomy) ≥6 weeks before screening. Participants must have been in generally good health as determined by review of medical history and physical exam, with documentation of a recent mammogram (obtained at screening or ≤9 months before enrollment) showing normal/negative or no clinically significant findings, and a body mass index of 18-38 kg/m^2^.

Participants were ineligible for the study if they had a history of any of the following: severe allergy/intolerance to drugs in general or any of the excipients in the study medication; drug or alcohol abuse; malignant tumor (except for basal cell carcinoma); endometrial hyperplasia or uterine/endometrial cancer; unexplained uterine bleeding; seizures or other convulsive disorders; suicide attempt (past 3 years); and any other medical condition, chronic disease, or malignancy that could confound interpretation of the study outcome and/or interfere with the absorption, distribution, metabolism, or excretion of the study drug. Additionally, participants were excluded if they had active liver disease or jaundice or elevated liver enzyme levels at screening (alanine aminotransferase [ALT] and aspartate aminotransferase [AST] > 1.5 times the upper limit of normal [ULN]; total bilirubin > 1.5 × ULN); creatinine > 1.5 × ULN; or estimated glomerular filtration rate ≤59 mL/min/1.73 m^2^. Other exclusion criteria included uncontrolled hypertension with a systolic blood pressure ≥140 mmHg and/or a diastolic blood pressure ≥90 mmHg; endometrial biopsy with evidence of hyperplasia or endometrial cancer or inadequate specimen at screening; and any findings from the physical exam, vital sign assessment, or 12-lead electrocardiogram (ECG) that led the investigator to consider the person unsuitable for participation.

Participants were excluded if they currently or recently used (and were unwilling to washout) a prohibited therapy that could interfere with the occurrence of VMS (eg, HT, hormonal contraceptive, VMS medication [prescription, over the counter, or herbal]). Antidepressant use was permitted if the dose had not changed within the 3 months before screening. Prohibited medications had to be washed out after consultation with the prescribing physician and as per product labeling, with a minimum washout period of five half-lives before screening visit. Any prior hormone therapy had to be discontinued for at least the following durations: ≥1 week for vaginal hormone products, ≥4 weeks for transdermal estrogen- and/or progestogen-containing products, ≥8 weeks for oral or intrauterine hormone products, ≥3 months for progestogen implants or estrogen injectable therapy, and ≥6 months for prior estrogen pellet therapy or progestogen injectable therapy. All therapies (prescription, over the counter, and herbal) administered ≤90 days before informed consent were recorded at screening and coded with the World Health Organization (WHO) Drug Dictionary (Mar 2017E B2). Previous use of VMS medications is summarized by therapeutic/chemical subgroups and preferred WHO drug name by treatment group.

### Study endpoints and assessments

#### Efficacy

Coprimary endpoints were mean change in the frequency of moderate/severe VMS from baseline to week 4; mean change in the frequency of moderate/severe VMS from baseline to week 12; mean change in the severity of moderate/severe VMS from baseline to week 4; and mean change in the severity of moderate/severe VMS from baseline to week 12.

Secondary efficacy endpoints included mean changes and percent reductions in VMS frequency and severity from baseline to each study week and the proportion of participants achieving a 50% reduction in moderate/severe VMS over time. Additional secondary endpoints included analyzing VMS outcomes for mild, moderate, and severe VMS; patient-reported outcomes; hot flash scores; and alternative definitions of response, which will be reported separately.

Participants recorded their VMS at least twice daily (morning and evening). Mild VMS were defined as sensations of heat without sweating or noting damp sheets or clothing upon awakening.^33,34^ Moderate VMS were defined as sensations of heat with sweating but being able to continue activities or waking from sleep because of feeling hot.^33,34^ Severe VMS were defined as feelings of intense heat with sweating that disrupts activities or, for night sweats, feelings of being so hot as to require action (eg, remove layers of clothing, open a window).^33,34^

Data from VMS recordings collected during the 35-day screening period were used to determine participant eligibility on the basis of VMS frequency and severity. Study baseline values were calculated based on mean VMS frequency and severity recorded over the last seven consecutive calendar days with nonmissing data before day 1 of the treatment period. As a consequence, the baseline could be less than the required 50 moderate/severe VMS per week study entry criterion, since eligibility could have been established anytime during the 35-day screening period.

VMS frequency was counted by the number of moderate or severe VMS in a 24-hour period. The moderate/severe VMS severity per day was determined by the following calculation: [(number of moderate VMS × 2) + (number of severe VMS × 3)]/(number of moderate + number of severe VMS). For participants with no moderate or severe VMS, the severity was calculated as 0; weekly average severity was calculated as the mean of daily severity scores over seven days.

#### Pharmacodynamics

Plasma concentrations of luteinizing hormone (LH), FSH, estradiol, and sex hormone-binding globulin (SHBG) were measured as prespecified pharmacodynamic (PD) endpoints. Blood samples for PD evaluation were taken before the morning dose on day 1 and at weeks 4, 8, and 12; 3 hours after the morning dose at week 4 or in some cases at week 8 or 12 according to participant availability; and at the follow-up visit. LH, FSH, estradiol, and SHBG concentrations were analyzed by electrochemiluminescence (Roche cobas e 601 module). The measurement range was 0.1 to 200.0 IU/L for LH and FSH, 73.4 to 11,013 pmol/L for estradiol, and 3.59 to 200.0 nmol/L for SHBG. Women with an estradiol level below the limit of quantification (73.4 pmol/L) were imputed as having a result at half that limit (ie, 36.7 pmol/L).

Participants fasted for ≥10 hours before study visits that included PD assessments and through at least 1 hour postdose.

#### Safety

Assessments of safety and tolerability included treatment-emergent AEs (TEAEs) monitoring and laboratory testing. Additional safety measures included transvaginal ultrasound with endometrial biopsy if required and at end of treatment, vital signs, ECG parameters, and plasma bone turnover markers consisting of bone alkaline phosphatase, procollagen type 1 amino-terminal propeptide, and carboxy-terminal telopeptide of type I collagen.

### Statistical analyses

Sample size was based on treatment effect size from the preceding phase 2a trial in which fezolinetant 90 mg BID reduced the frequency of moderate/severe VMS by about 5.0 (95% confidence interval [CI]: −6.8 to −3.3) per day relative to placebo. It was estimated that 40 participants per treatment group would have > 80% power to detect a difference of 3.3 VMS/day for any given pairwise comparison using a 2-sample *t* test at a 2-sided 5% alpha. Similarly, in the phase 2a study, fezolinetant reduced severity of moderate/severe VMS by 1.12 (95% CI: −1.5 to −0.74) relative to placebo, so a sample size of 40 was estimated to provide > 80% power to detect a difference in severity of 0.64 points for similar pairwise comparisons. Combined power to test for all four coprimary endpoints was lower than the power to test for each endpoint individually. To allow for up to a 10% dropout rate, planned enrollment was 44 participants per treatment arm, for a total of 352 randomized participants.

The safety population included all participants who received at least one dose of study treatment. Efficacy analyses were reported for the full analysis set, which comprised participants who received at least one dose of study drug and had at least one postbaseline efficacy evaluation.

For each of the coprimary efficacy endpoints, an analysis of covariance (ANCOVA) model was used with treatment group, pooled centre, and smoking status (current vs former/never) as factors, with baseline weight and baseline measurement as covariates. Pairwise comparisons between the active doses and placebo were calculated based on least squares mean contrasts using a two-sided test at 5% error rate without the multiplicity adjustment. Missing primary efficacy endpoints were imputed using multiple imputations by fully conditional specification methods.

Odds of response (50% reduction in moderate/severe VMS frequency at last on-treatment assessment) were calculated for fezolinetant versus placebo based on logistic regression analysis, with treatment group and smoking status as factors and baseline frequency of VMS as a covariate. Nonresponder imputation was used for missing response data. Change in mean frequency and severity of VMS per 24 hours was analyzed for each week using a mixed effect model for repeated measures, with change from baseline as the dependent variable and treatment group, visit, and smoking status as factors and baseline measurement as a covariate, as well as interaction of treatment by week and an interaction of baseline measurement by week. PD results were analyzed using descriptive statistics.

Statistical analyses were performed using SAS software, version 9.3 or higher.

## RESULTS

### Study population

**FIG. 1:**
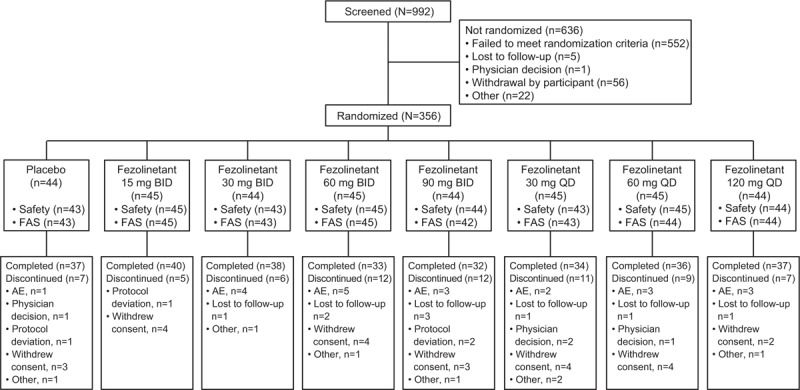
Participant disposition. Safety included all participants who were randomized and received at least one dose of study medication. FAS included all randomized participants who received at least one dose of study drug and had baseline and postbaseline efficacy evaluation. AE, adverse event; FAS, full analysis set.

Of 992 participants who were screened, 356 were randomly allocated to receive placebo or one of the seven fezolinetant regimens (Fig. 1). A total of 352 participants received study medication and were included in the safety population, 349 were included in the full analysis set, and 287 (80.6%) completed the 12-week study period. Withdrawal of consent (6.7%) and AEs (5.9%) were the most common reasons for premature study discontinuation.

Participants ranged in age from 41 to 65 years (mean: 54.6 y), and the study population was ∼73% white, 25% black, 1% Asian, and 1% other races. Baseline demographics were similar across treatment groups (Table 1). At baseline, participants had an average of 9 to 11 moderate/severe VMS per day, which was similar across treatment groups. Mean (SD) estradiol levels ranged from 46.1 (26.3) to 73.7 (153.8) pmol/L across the treatment groups at baseline.

Use of previous medications was recorded for 56 participants (15.9%). Use of previous treatments for VMS is summarized (Table 2). A total of 15 participants (4.3%) had been previously treated with hormone therapy (therapeutic subgroup: sex hormones and modulators of the genital system), and two participants had previously received a nonhormonal treatment (paroxetine).

### Primary VMS efficacy outcomes

**FIG. 2:**
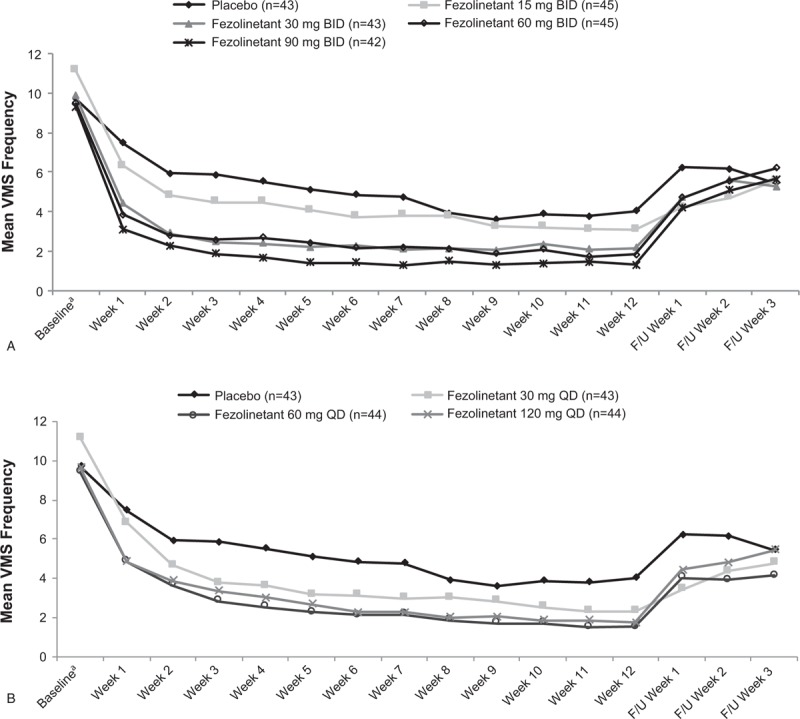
Frequency of moderate and severe VMS per 24 hours with fezolinetant BID (**A**) or Fezolinetant QD dosing (**B**), Full analysis set. F/U, follow-up; VMS, vasomotor symptoms. ^*a*^Baseline is the average frequency of 24-hour VMS from seven nonmissing days before day 1.

**FIG. 3:**
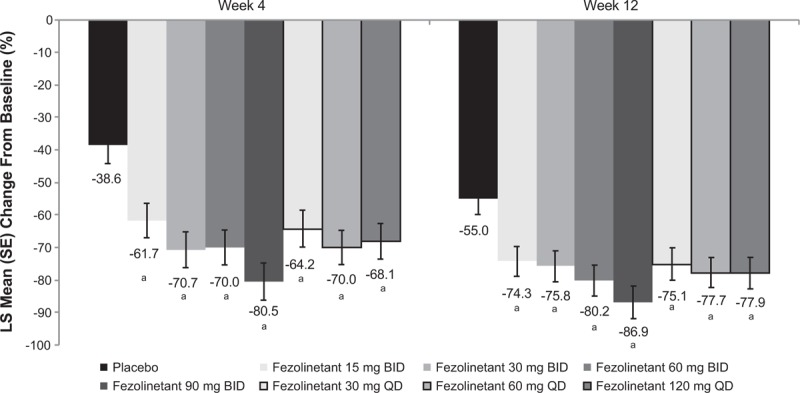
LS mean percentage reduction from baseline in frequency of moderate/severe VMS (MMRM), full analysis set. MMRM conducted with treatment group, visit, and smoking status as factors, and baseline measurement, interaction of treatment by week, and interaction of baseline measurement by week as covariates. LS, least squares; MMRM, mixed effect model for repeated measures; VMS, vasomotor symptoms. ^*a*^*P < *0.05 for all pairwise comparisons of fezolinetant versus placebo at weeks 4 and 12, with no adjustments for multiplicity.

All treatment groups exhibited a decrease in frequency of moderate/severe VMS (Fig. 2). All fezolinetant regimens significantly reduced the frequency of moderate/severe VMS at weeks 4 and 12 based on pairwise comparisons with placebo (Table 3). Frequency was reduced by more than two moderate/severe VMS per day relative to placebo at weeks 4 and 12 for all fezolinetant dose groups except 15 mg BID (Table 3). Fezolinetant reduced moderate/severe VMS by about 62% to 81% at week 4, depending on dose, compared with about a 39% reduction with placebo; at week 12, moderate/severe VMS were reduced by about 74% to 87% with fezolinetant versus 55% with placebo (Fig. 3).

**FIG. 4:**
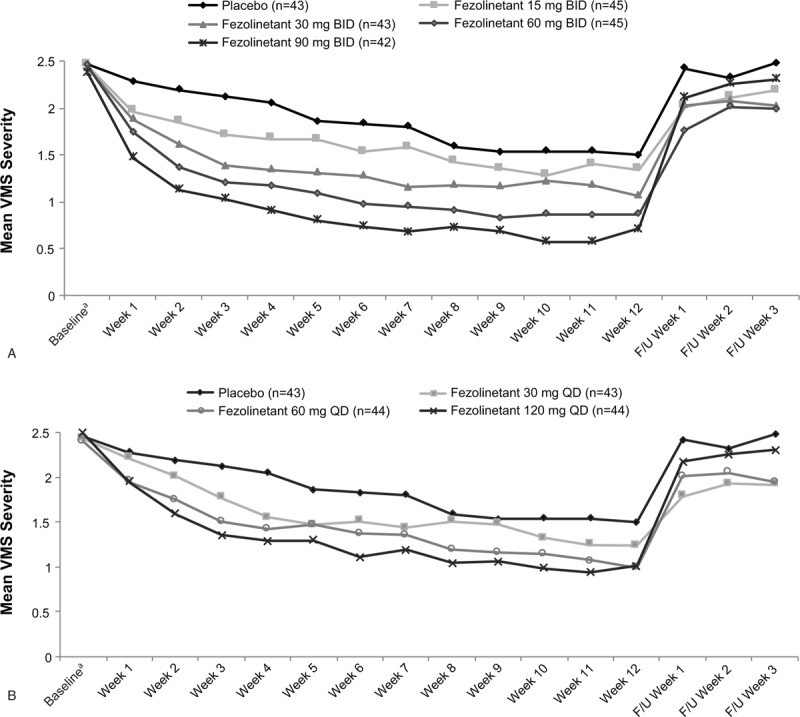
Severity of moderate and severe VMS per 24 hours with fezolinetant BID (**A**) or fezolinetant QD dosing (**B**), Full analysis set. F/U, follow-up; VMS, vasomotor symptoms. ^*a*^Baseline is the average severity of 24-hour VMS from seven nonmissing days before day 1.

All fezolinetant BID (Fig. 4A) and QD (Fig. 4B) treatment groups and the placebo group had decreases from baseline in severity of moderate/severe VMS. In the primary analysis, all fezolinetant regimens significantly reduced the severity of moderate/severe VMS relative to placebo at week 4, and fezolinetant 60 mg BID, 90 mg BID, and 60 mg QD significantly reduced VMS severity relative to placebo at week 12 (Table 3). Least squares mean differences from placebo in severity of moderate/severe VMS were −0.4 to −1 at week 4 and −0.2 to −0.6 at week 12 across all fezolinetant regimens.

### Secondary VMS efficacy outcomes

Week-by-week analysis showed that all fezolinetant regimens decreased the frequency (Fig. 2A and B) and severity (Fig. 4A and B) of moderate/severe VMS from the first week through the last week of treatment, with a return of symptoms after discontinuation. Early reductions in frequency and severity during weeks 1 and 2 were confirmed via mixed effect model for repeated measures analysis for a majority of doses (Supplemental Digital Content Table S1). Across all fezolinetant regimens studied, approximately 81% to 95% of participants achieved ≥50% reductions in frequency of VMS by last on-treatment assessment, compared with about 59% in the placebo group. Participants who received fezolinetant were 3.2 to 12.7 times as likely (based on odds ratios) to achieve a 50% reduction in moderate/severe VMS by last on-treatment assessment compared with those taking placebo (Supplemental Digital Content Figure S1).

When data were analyzed for all hot flashes of any severity (mild, moderate, and severe), results were similar to those for moderate/severe VMS (Supplemental Digital Content Table S2). At week 4, all fezolinetant doses significantly reduced VMS frequency and severity versus placebo. At week 12, all fezolinetant doses except 15 mg BID significantly reduced frequency of VMS of any severity and all doses except 15 mg BID and 30 mg QD significantly reduced severity of VMS. Odds of response based on 50% reduction in frequency of mild, moderate, and severe VMS at last on-treatment visit were significantly greater for all but the lowest two fezolinetant doses (15 mg BID and 30 mg QD) versus placebo.

### PD outcomes

Although LH levels remained relatively stable in the placebo group, fezolinetant was associated with dose-dependent LH reductions 3-hours postdose compared with either baseline or predose. All groups, including placebo, showed small decreases from baseline in FSH levels, and no treatment- or dose-related effects were observed. There were no clear trends or differences from placebo in estradiol or SHBG levels over the course of the study. A majority of women in this postmenopausal population had estradiol levels below the limit of quantification and were imputed as having a result at half that limit (36.7 pmol/L); this led to a skewing of the summary statistics such that the median value was 36.7 pmol/L for all treatment groups.

### Safety

Rates of TEAEs were similar across treatment groups (Table 4), with no indication of a dose effect. The most common TEAEs were nausea, diarrhea, fatigue, urinary tract infection, upper respiratory tract infections, sinusitis, headache, and cough. There was one serious TEAE consisting of squamous cell carcinoma of the skin in a woman treated with fezolinetant 60 mg QD who had a skin lesion before study enrollment; this TEAE was not considered treatment related. There were no deaths during the study period. Twenty-one participants discontinued because of AEs (Table 4); the only TEAEs leading to permanent treatment discontinuation in more than one participant were elevated liver enzymes (*n *= 5), vertigo (*n *= 2), headache (*n *= 2), and depression (*n *= 2).

TEAEs were mild or moderate, except for five severe TEAEs: liver function test values increased (*n *= 1, fezolinetant 90 mg BID), adjustment disorder with depressed mood (*n *= 1, fezolinetant 30 mg QD), cholelithiasis (*n *= 1, fezolinetant 60 mg QD), drug-induced liver injury (*n *= 1, fezolinetant 60 mg QD), and retinal detachment (*n *= 1, fezolinetant 120 mg QD). The cholelithiasis and drug-induced liver injury were considered treatment-related events by the investigator. The TEAE reported as drug-induced liver injury consisted of asymptomatic elevations in ALT (14.1 × ULN) and AST (9.5 × ULN) in a participant with obesity (BMI: 32 kg/m^2^) and nonalcoholic steatohepatitis; ALT/AST levels normalized after treatment discontinuation.

Potentially clinically relevant liver function test results are shown in Supplemental Digital Content Table S3. Nine participants had ALT or AST >3 × ULN, and three of these participants (one each in the fezolinetant 60 mg BID, 90 mg BID, and 60 mg QD groups) had ALT or AST >8 × ULN. None of the participants developed total bilirubin >2 × ULN; consequently, no cases met the criteria for Hy's law. ALT/AST levels rapidly returned to baseline values after discontinuation of treatment; two participants who did not immediately discontinue therapy demonstrated a clear trend toward normalization of ALT/AST levels while maintained on study drug. Five participants (one each in the placebo, fezolinetant 15 mg BID, 90 mg BID, 30 mg QD, and 120 mg QD groups) transiently developed creatine kinase levels >1000 U/L; their levels returned to normal at subsequent visits.

There were no clinically meaningful changes in vital signs and no clinically meaningful changes over time or differences from placebo in ECG results with fezolinetant. Changes in bone turnover markers were no different across treatment groups. Five participants experienced uterine bleeding during the study, including one (2.3%) in the placebo group, one (2.3%) in the fezolinetant 30 mg BID group, one (2.2%) in the fezolinetant 60 mg BID group, and two (4.7%) in the fezolinetant 30 mg QD group. There were no clinically significant endometrial biopsy findings and no meaningful changes from baseline or compared with placebo in endometrial thickness measured by transvaginal ultrasound (Supplemental Digital Content Figure S2; median change −1.0 to 1 mm across fezolinetant dose groups versus 0 mm in the placebo group).

## DISCUSSION

This study evaluated the effect of seven fezolinetant doses and dosing regimens on the frequency and severity of VMS associated with menopause. Multiple dose levels and both once- and twice-daily dosing regimens of fezolinetant reduced the frequency and severity of VMS, as early as the first week with most doses. Findings were consistent with those from the previous phase 2a trial, in which fezolinetant produced significant reductions in VMS frequency and severity in VMS associated with menopause and was well tolerated.^32^

In the current study, all fezolinetant doses except the lowest dose (15 mg BID) resulted in reductions of more than two moderate/severe VMS per day at weeks 4 and 12, relative to placebo. Reductions of more than two VMS/day compared with placebo are generally recognized by the FDA as clinically relevant.^35,36^ In addition, 81% to 95% of participants treated with fezolinetant experienced a 50% reduction in the frequency of moderate/severe VMS, a relative magnitude of change that has been reported to be clinically meaningful.^37,38^

The rapid, substantial reduction in VMS frequency and severity observed with fezolinetant is consistent with previous results reported for other NK3R antagonists, MLE4901(formerly in development)^39,40^ and the mixed NK1R/NK3R antagonist NT-814.^41^ Thus, the current study adds to the growing body of literature describing the neural circuits in the hypothalamus underlying the loss of thermoregulatory control in VMS and further exemplifies the importance of NK3R signaling as a therapeutic target for the nonhormone treatment of hot flashes. Our findings and those of others demonstrate that NK3R antagonists improve VMS without altering plasma levels of ovarian hormones such as estradiol in menopausal women.^32,39^ Indeed, baseline estradiol and gonadotropin levels are consistent with those of generally healthy menopausal women,^32^ and no change in estradiol was observed over the course of the study. Some dose-dependent suppression of LH was observed, but this was not linked to any changes in estradiol, and is consistent with the known, centrally acting effects of NK3 receptor antagonists.

In the current study, we extend previous findings by demonstrating that fezolinetant was not associated with an increase in the thickness of the endometrial lining or endometrial hyperplasia (a risk factor for endometrial cancer), which has been reported with the use of unopposed estrogens as part of HT for VMS.^42^ Therefore, fezolinetant as a novel, nonhormone, centrally acting drug may provide an option for women who desire treatment for VMS but have contraindications to or concerns regarding the use of hormone products.

Fezolinetant was well tolerated. Overall, TEAE rates were similar across treatment groups and were mostly mild and moderate. No deaths or treatment-related serious AEs were reported. Nine participants experienced transient ALT or AST elevations >3 × ULN, typically between 4 and 8 weeks of treatment. There were no cases of bilirubin > 2 × ULN and therefore no Hy's law cases (ie, ALT or AST > 3 × ULN + total bilirubin > 2 × ULN without cholestasis and without alternative explanation), which is used by the FDA to identify drugs likely to cause severe drug-induced liver injury.^43^ The observed elevations in ALT/AST were transient regardless of treatment duration; levels returned to baseline following discontinuation of treatment and trended toward baseline values in those participants who remained on treatment. Importantly, none of these elevations were associated with evidence of liver functional impairment or liver-associated symptoms. The safety profile of fezolinetant shall be fully characterized in phase 3 clinical trials.

One limitation of this study was that participants were eligible for inclusion if they recorded ≥50 moderate/severe VMS over any seven consecutive days during the 35-day screening period; therefore, not all of the participants had ≥50 moderate/severe VMS during the week immediately preceding randomization. In addition, since participants who were receiving other treatments that may have affected VMS or who had disorders that might have interfered with interpretation of the study were excluded, results may not be generalizable to all menopausal women. Other limitations of this phase 2 study include the short duration of treatment (12 wk), small number of participants per treatment arm (*n *= 43-45 per group), and lack of adjustments for multiplicity.

## CONCLUSIONS

Results of this study suggest that fezolinetant is a well-tolerated nonhormone therapy that produces rapid reduction of moderate/severe VMS associated with menopause. Efficacy was demonstrated at multiple dose levels and with both once- and twice-daily administration, and efficacy was observed by the first week with some doses. Further evaluation of fezolinetant in larger and longer phase 3 trials of women with VMS associated with menopause is warranted to more fully characterize its efficacy and safety profile.

## Supplementary Material

Supplemental Digital Content

## Supplementary Material

Supplemental Digital Content

## Supplementary Material

Supplemental Digital Content

## Figures and Tables

**TABLE 1:** Baseline demographics and clinical characteristics^*a*^

Parameters	Placebo (*n *= 43)	Fezolinetant						
		15 mg BID (*n *= 45)	30 mg BID (*n *= 43)	60 mg BID (*n *= 45)	90 mg BID (*n *= 44)	30 mg QD (*n *= 43)	60 mg QD (*n *= 45)	120 mg QD (*n *= 44)
Age, y, mean (SD)	54.8 (5.5)	53.7 (5.0)	53.9 (3.8)	54.6 (5.0)	54.9 (4.0)	52.7 (3.8)	55.0 (4.9)	56.8 (4.4)
BMI, kg/m^2^, mean (SD)	27.3 (4.8)	29.3 (4.3)	28.3 (4.0)	29.1 (5.2)	27.3 (4.6)	28.8 (4.0)	28.3 (4.4)	28.8 (4.9)
Race, *n* (%)								
White	30 (69.8)	37 (82.2)	31 (72.1)	28 (62.2)	36 (81.8)	31 (72.1)	34 (75.6)	30 (68.2)
African American	10 (23.3)	8 (17.8)	12 (27.9)	15 (33.3)	8 (18.2)	11 (25.6)	10 (22.2)	13 (29.5)
Asian	2 (4.7)	0	0	1 (2.2)	0	0	0	0
Other	1 (2.3)	0	0	1 (2.2)	0	1 (2.3)	1 (2.2)	1 (2.3)
Ethnicity, *n* (%)								
Hispanic/Latino	15 (34.9)	16 (35.6)	9 (20.9)	13 (28.9)	10 (22.7)	17 (39.5)	12 (26.7)	9 (20.5)
Not Hispanic/Latino	28 (65.1)	29 (64.4)	34 (79.1)	32 (71.1)	34 (77.3)	26 (60.5)	33 (73.3)	35 (79.5)
Smoking status, *n* (%)								
Current	3 (7.0)	10 (22.2)	5 (11.6)	8 (17.8)	4 (9.1)	3 (7.0)	11 (24.4)	3 (6.8)
Former	6 (14.0)	7 (15.6)	12 (27.9)	8 (17.8)	12 (27.3)	12 (27.9)	11 (24.4)	5 (11.4)
Never	34 (79.1)	28 (62.2)	26 (60.5)	29 (64.4)	28 (63.6)	28 (65.1)	23 (51.1)	36 (81.8)
Type of menopause, *n* (%)								
Natural	25 (58.1)	27 (60.0)	35 (81.4)	28 (62.2)	32 (72.7)	27 (62.8)	36 (80.0)	35 (79.5)
Baseline moderate/severe VMS, mean (SD)^*b*^^,^^*c*^								
Frequency/24 h	9.7 (3.5)	11.1 (7.1)	9.9 (4.6)	9.5 (4.0)	9.3 (3.6)	11.2 (6.4)	9.4 (2.7)	9.7 (3.7)
Severity/24 h	2.5 (0.3)	2.5 (0.3)	2.4 (0.3)	2.5 (0.3)	2.4 (0.3)	2.4 (0.3)	2.4 (0.3)	2.5 (0.3)
Estradiol, pmol/L, mean (SD)^*b*^^,^^*d*^^,^^*e*^	48.7 (49.4)	53.3 (88.4)	53.9 (64.7)	53.6 (55.5)	59.5 (99.6)	73.7 (153.8)	68.2 (197.1)	46.1 (26.3)
FSH, IU/L, mean (SD)^*b*^^,^^*e*^	74.1 (27.7)	75.7 (26.3)	85.2 (30.9)	75.2 (34.6)	71.6 (24.1)	80.2 (29.4)	76.4 (27.7)	81.9 (25.3)
LH, IU/L, mean (SD)^*b*^^,^^*e*^	39.2 (13.7)	40.1 (13.3)	43.7 (12.9)	41.0 (18.0)	39.3 (13.3)	44.3 (12.6)	42.9 (18.5)	43.3 (12.5)
SHBG, nmol/L, mean (SD)^*b*^^,^^*e*^	74.7 (37.8)	58.5 (36.5)	60.6 (33.7)	71.5 (57.7)	69.1 (37.0)	65.8 (42.9)	68.9 (38.6)	76.6 (40.1)

BMI, body mass index; FSH, follicle stimulating hormone; LH, luteinizing hormone; SD, standard deviation; SHBG, sex hormone-binding globulin; VMS, vasomotor symptoms.

^*a*^Safety population, unless otherwise specified.

^*b*^Values are from the full analysis set.

^*c*^Baseline is average frequency/severity of 24-hour VMS from seven nonmissing days before day 1.

^*d*^The value for estradiol was imputed as 73.4/2 = 36.7 pmol/L when result was < 73.4 pmol/L.

^*e*^The *n*'s for these measurements include only those participants with nonmissing values at baseline.

**TABLE 2:** Prior exposure to medications for VMS

Medication	Placebo (*n *= 43)	Fezolinetant						
		15 mg BID (*n *= 45)	30 mg BID (*n *= 43)	60 mg BID (*n *= 45)	90 mg BID (*n *= 44)	30 mg QD (*n *= 43)	60 mg QD (*n *= 45)	120 mg QD (*n *= 44)
Sex hormones and modulators of the genital system, *n* (%)^*a*^	4 (9.3)	1 (2.2)	1 (2.3)	0	3 (6.8)	2 (4.7)	3 (6.7)	1 (2.3)
Estrogen and/or progestogen therapy, *n* (%)								
Estradiol	2 (4.7)	0	1 (2.3)	0	0	2 (4.7)	1 (2.2)	0
Estradiol benzoate	0	0	0	0	1 (2.3)	0	0	0
Conjugated estrogens	2 (4.7)	0	0	0	1 (2.3)	0	0	0
Progesterone	0	0	0	0	0	0	1 (2.2)	0
Estrogen/progestogen combinations	0	0	0	0	1 (2.3)	0	1 (2.2)	0
Other^*b*^	1 (2.3)	1 (2.2)	0	0	0	0	1 (2.2)	1 (2.3)
SSRIs (paroxetine or paroxetine mesylate), *n* (%)	1 (2.3)	0	0	0	0	0	0	1 (2.3)

SSRI, selective serotonin reuptake inhibitor; VMS, vasomotor symptoms.

^*a*^Total *N* for the category in each treatment group may be less than the sum of the *n*'s for the individual medications as participants may have taken more than one medication.

^*b*^Includes herbal treatments with estrogen-like activity (cimicifuga racemosa extract) and chorionic gonadotrophin.

**TABLE 3:** Primary efficacy outcomes: frequency of moderate/severe VMS and severity per 24 hours, full analysis set

		Frequency of moderate/severe VMS per 24 h^*a*^				Severity of moderate/severe VMS per 24 h^*a*^			
		Change from baseline	Difference from placebo			Change from baseline	Difference from placebo		
Wk	Treatment group (n)	LS Mean (SE)	LS Mean (SE)	95% CI	*P* value^*b*^	LS Mean(SE)	LS Mean (SE)	95% CI	*P* value^*b*^
4	Placebo (*n *= 42)	−4.2 (0.65)	–	–	–	−0.3 (0.15)	–	–	–
	Fezolinetant								
	15 mg BID (*n *= 40)	−6.1 (0.61)	−1.9 (0.84)	−3.56 to −0.25	0.024	−0.8 (0.14)	−0.5 (0.2)	−0.84 to −0.07	0.0215
	30 mg BID (*n *= 41)	−7.2 (0.64)	−3 (0.84)	−4.68 to −1.38	0.0004	−0.9 (0.15)	−0.6 (0.2)	−1.01 to −0.24	0.0017
	60 mg BID (*n *= 40)	−7.0 (0.62)	−2.8 (0.84)	−4.44 to −1.14	0.0010	−1.2 (0.14)	−0.8 (0.2)	−1.21 to −0.44	<0.0001
	90 mg BID (*n *= 37)	−7.7 (0.65)	−3.5 (0.84)	−5.20 to −1.89	<0.0001	−1.3 (0.15)	−1 (0.2)	−1.37 to −0.59	<0.0001
	30 mg QD (*n *= 40)	−6.5 (0.65)	−2.3 (0.84)	−4.00 to −0.68	0.0058	−0.7 (0.15)	−0.4 (0.2)	−0.81 to −0.04	0.0322
	60 mg QD (*n *= 43)	−7.2 (0.61)	−3 (0.82)	−4.65 to −1.41	0.0003	−0.9 (0.14)	−0.6 (0.19)	−0.99 to −0.23	0.0017
	120 mg QD (*n *= 42)	−6.6 (0.63)	−2.4 (0.84)	−4.06 to −0.76	0.0042	−1.0 (0.15)	−0.7 (0.2)	−1.08 to −0.31	0.0004
12	Placebo (*n *= 37)	−5.3 (0.58)	–	–	–	−0.8 (0.16)	–	–	–
	Fezolinetant								
	15 mg BID (*n *= 38)	−7.2 (0.54)	−1.8 (0.75)	−3.30 to −0.35	0.0154	−1.0 (0.15)	−0.3 (0.21)	−0.67 to 0.16	0.2324
	30 mg BID (*n *= 37)	−7.5 (0.56)	−2.1 (0.74)	−3.60 to −0.67	0.0043	−1.1 (0.16)	−0.4 (0.21)	−0.80 to 0.04	0.0736
	60 mg BID (*n *= 31)	−7.6 (0.55)	−2.3 (0.75)	−3.76 to −0.83	0.0023	−1.3 (0.16)	−0.6 (0.21)	−0.98 to −0.15	0.0080
	90 mg BID (*n *= 31)	−8.0 (0.58)	−2.6 (0.75)	−4.09 to −1.16	0.0005	−1.4 (0.17)	−0.6 (0.21)	−1.07 to −0.22	0.0028
	30 mg QD (*n *= 33)	−7.4 (0.58)	−2.1 (0.75)	−3.52 to −0.58	0.0064	−0.9 (0.16)	−0.2 (0.21)	−0.58 to 0.26	0.4647
	60 mg QD (*n *= 36)	−7.9 (0.54)	−2.6 (0.74)	−4.04 to −1.15	0.0005	−1.3 (0.15)	−0.5 (0.21)	−0.92 to −0.10	0.0160
	120 mg QD (*n *= 36)	−7.4 (0.57)	−2.1 (0.75)	−3.52 to −0.59	0.0063	−1.1 (0.16)	−0.4 (0.21)	−0.78 to 0.06	0.0901

ANCOVA, analysis of covariance; LS, least squares; SE, standard error; VMS, vasomotor symptoms.

^*a*^From ANCOVA model with change from baseline as the dependent variable and treatment group, pooled centre, smoking status as factors and baseline measurement and baseline weight as covariates.

^*b*^Based on pairwise comparisons with placebo without adjustments for multiplicity.

**TABLE 4:** Treatment-emergent adverse events, safety analysis set

		Fezolinetant, *n* (%)						
	Placebo (*n *= 43), n (%)	15 mg BID (*n *= 45)	30 mg BID (*n *= 43)	60 mg BID (*n *= 45)	90 mg BID (*n *= 44)	30 mg QD (*n *= 43)	60 mg QD (*n *= 45)	120 mg QD (*n *= 44)
TEAEs	21 (48.8)	20 (44.4)	18 (41.9)	21 (46.7)	19 (43.2)	23 (53.5)	28 (62.2)	22 (50.0)
Serious TEAEs	0	0	0	0	0	0	1 (2.2)^*a*^	0
TEAEs leading to permanent discontinuation	1 (2.3)	0	4 (9.3)	5 (11.1)	3 (6.8)	2 (4.7)	3 (6.7)	3 (6.8)
TEAEs leading to treatment interruption	1 (2.3)	0	0	2 (4.4)	3 (6.8)	0	0	1 (2.3)
Any treatment-related TEAEs	3 (7.0)	1 (2.2)	9 (20.9)	8 (17.8)	9 (20.5)	10 (23.3)	12 (26.7)	11 (25.0)
TEAEs occurring in ≥5% in any treatment arm								
Headache	2 (4.7)	3 (6.7)	2 (4.7)	2 (4.4)	1 (2.2)	6 (14.0)	3 (6.7)	4 (9.1)
Nausea	1 (2.3)	1 (2.2)	3 (7.0)	3 (6.7)	1 (2.3)	2 (4.7)	3 (6.7)	2 (4.5)
Urinary tract infection	1 (2.3)	2 (4.4)	1 (2.3)	2 (4.4)	2 (4.5)	2 (4.7)	2 (4.4)	3 (6.8)
Diarrhea	1 (2.3)	0	1 (2.3)	2 (4.4)	2 (4.5)	1 (2.3)	3 (6.7)	2 (4.5)
Upper respiratory tract infection	1 (2.3)	2 (4.4)	1 (2.3)	1 (2.2)	1 (2.3)	3 (7.0)	1 (2.2)	1 (2.3)
Fatigue	0	1 (2.2)	1 (2.3)	1 (2.2)	2 (4.5)	0	3 (6.7)	1 (2.3)
Viral upper respiratory tract infection	0	2 (4.4)	1 (2.3)	1 (2.2)	3 (6.8)	0	0	0
Sinusitis	0	0	0	3 (6.7)	0	1 (2.3)	2 (4.4)	0
Cough	0	1 (2.2)	0	1 (2.2)	0	0	3 (6.7)	0

TEAE, treatment-emergent adverse event.

^*a*^Skin squamous cell carcinoma in a participant who had a preexisting skin mass; not considered treatment related.
