# Supplementary material for: A phase 2b, randomized, placebo-controlled, double-blind, dose-ranging study of the neurokinin 3 receptor antagonist fezolinetant for vasomotor symptoms associated with menopause
Source: Menopause. 2020 Feb 24;27(4):382–92. doi: 10.1097/GME.0000000000001510 (PMC7147405; doi:10.1097/GME.0000000000001510)
Supplement: Supplemental Digital Content [file menop-27-382-s002.docx]

**Script for Fezolinetant 205 primary video abstract for *Menopause***

**Manuscript title:** a Phase 2b, Randomized, Placebo-Controlled, Double-Blind, Dose-Ranging Study of the Neurokinin 3 Receptor Antagonist Fezolinetant for Vasomotor Symptoms associated with Menopause

Graeme L. Fraser, PhD^1^; Samuel Lederman, MD^2^; Arthur Waldbaum, MD^3^; Robin Kroll, MD^4^; Nanette Santoro, MD^5^; Misun Lee, PhD^6^; Laurence Skillern, MD^7^; Steven Ramael, MD^1^

^1^OGEDA SA, a wholly owned subsidiary of Astellas Pharma SA, Brussels, Belgium (graeme.fraser@astellas.com; steven.ramael@telenet.be); ^2^Altus Research, Lake Worth, FL, USA ([sleder@altusresearch.com](mailto:sleder@altusresearch.com)); ^3^Downtown Women’s Healthcare, Denver, CO, USA ([DrWaldbaum@md4women.com](mailto:DrWaldbaum@md4women.com)); ^4^Seattle Women’s: Health, Research, Gynecology®, Seattle, WA, USA ([rkroll@seattlewomens.com](mailto:rkroll@seattlewomens.com)); ^5^University of Colorado School of Medicine, Aurora, CO, USA ([nanette.santoro@ucdenver.edu](mailto:nanette.santoro@ucdenver.edu)); ^6^Astellas Pharma, Inc., Northbrook, IL, USA ([Misun.Lee@Astellas.com](mailto:Misun.Lee@Astellas.com)); ^7^Astellas Pharma Europe Ltd, Chertsey, UK ([laurenceskillern@talktalk.net](mailto:laurenceskillern@talktalk.net))

**Audio**

My name is Graeme Fraser. I was formerly the chief scientific officer of OGEDA, a wholly owned subsidiary of Astellas Pharma.

On behalf of my coauthors, I'm pleased to present highlights from VESTA, a phase 2b study of fezolinetant as a treatment for vasomotor symptoms associated with menopause

Vasomotor symptoms, otherwise known as hot flashes and night sweats, are reported by up to 80% of US women during menopause. And they persist for a median period of about 7 years.

Vasomotor symptoms are caused by a loss of thermoregulatory control. In premenopausal women, estrogen has a role in homeostatic, negative feedback of certain neurons (known as KNDy neurons) in the brain that play a role in thermoregulatory control. But in menopause, estrogen declines and its inhibitory effect on these neurons is lost. The neurokinin 3 NK3 receptor is also expressed on KNDy neurons, and it is now understood that antagonism of the NK3 receptor is another way of controlling the firing of those neurons. This is the scientific approach that we've taken to try and treat vasomotor symptoms

Today I’ll be talking about fezolinetant, an antagonist to the NK3 receptor

Previously we evaluated fezolinetant at a dose of 90 mg twice daily in the treatment of vasomotor symptoms in a proof-of-concept, double-blind, placebo-controlled trial, which is now published.

Today I'll be discussing the testing of additional doses and dose regimens to build on that previous data

The objectives of the current study were to evaluate the efficacy of various dose levels of fezolinetant on VMS frequency and severity and to assess the safety across a larger study population.

So this study was a phase 2b, randomized, placebo-controlled, patient- and investigator-blinded, dose-ranging, parallel group clinical trial conducted in the US in a population of 356 patients.

These were all postmenopausal women, aged 40 to 65 years, in good general health who had at least 50 moderate or severe vasomotor symptoms per week.

We tested a total of 7 different treatment groups along with a placebo control. The groups included various once-daily and twice-daily dose regimens, up to 90 mg twice daily.

The coprimary outcomes of this clinical trial were the changes in the frequency and severity of moderate and severe vasomotor symptoms at weeks 4 and 12 of treatment.

At weeks 4 and 12, you can see that all active treatment groups tested showed significantly greater improvement in frequency of moderate to severe VMS relative to placebo. And you can see from the data shown on the screen that both once per day and twice per day dose regimens reduced the frequency of VMS.

The other primary endpoint for this study was VMS severity. As shown here in tabular format, all fezolinetant QD and BID dose groups demonstrated reduction in severity at both week 4 and 12, with statistically significant differences found for all doses at week 4 and at 60 and 90 mg twice daily and 60 mg once daily at week 12.

With regard to treatment-emergent adverse events, overall rates were similar across all groups, and events were mostly mild and moderate.

One event that I will point out is an elevation in liver function tests. Nine patients showed elevated liver enzymes, with either AST or ALT more than 3 times the upper limit of normal. Liver enzyme levels rapidly returned to baseline after discontinuation of dosing.

With regard to other safety parameters of interest, there were no effects on ECG parameters, vital signs, or plasma bone markers. An important point is that we observed no clear trends or differences from placebo in estradiol levels throughout the study. As measured by endometrial biopsy at the end of treatment, we found no clinically significant endometrial abnormalities. And there also were no meaningful changes in endometrial thickness as measured by transvaginal ultrasound.

So in conclusion, fezolinetant treatment met the coprimary endpoints to significantly reduce the frequency and severity of VMS at both weeks 4 and 12. Efficacy was demonstrated at multiple dose levels and with both once-daily and twice-daily administration. Adverse events were similar across all groups, and events were mostly mild and moderate.

Overall, these results demonstrate the potential of fezolinetant as a well-tolerated and effective nonhormonal treatment for reduction of moderate and severe menopausal VMS. These findings support the further evaluation of fezolinetant for the treatment of VMS associated with menopause. Phase 3 trials, which are now underway, will more fully characterize its efficacy and safety profile.
