# Supplementary material for: A phase 2b, randomized, placebo-controlled, double-blind, dose-ranging study of the neurokinin 3 receptor antagonist fezolinetant for vasomotor symptoms associated with menopause
Source: Menopause. 2020 Feb 24;27(4):382–92. doi: 10.1097/GME.0000000000001510 (PMC7147405; doi:10.1097/GME.0000000000001510)

**SUPPLEMENTAL DIGITAL CONTENT**

**a Phase 2b, Randomized, Placebo-Controlled, Double-Blind, Dose-Ranging Study OF THE NEUROKININ 3 RECEPTOR ANTAGONIST FEZOLINETANT FOR VASOMOTOR SYMPTOMS associated with menopause**

Graeme L. Fraser, PhD; Samuel Lederman, MD; Arthur Waldbaum, MD;
Robin Kroll, MD; Nanette Santoro, MD; Misun Lee, PhD; Laurence Skillern, MD;
Steven Ramael, MD

**CONTENTS**

**Table S1.** Secondary Efficacy Outcomes: Frequency of Moderate/Severe VMS and Severity per 24 Hours in Weeks 1 and 2, Full Analysis Set

**Table S2.** Secondary Efficacy Outcomes: Frequency of Mild/Moderate/Severe VMS and Severity per 24 Hours, Full Analysis Set

**Table S3.** Liver Function Tests, Safety Analysis Set

**Figure S1.** Response Rates and Odds of Response with Fezolinetant Versus Placebo Based on ≥50% Reduction in Frequency of Moderate/Severe VMS at Last On-Treatment Visit, Full Analysis Set

**Figure S2.** Endometrial Thickness, Safety Analysis Set

**Table S1.** Secondary Efficacy Outcomes: Frequency of Moderate/Severe VMS and Severity per 24 Hours in Weeks 1 and 2, Full Analysis Set

| **Wk** | **Treatment group (n)** | **Frequency of moderate/severe VMS per 24 hours^a^** | | | | **Severity of moderate/severe VMS per 24 hours^a^** | | | |
| --- | --- | --- | --- | --- | --- | --- | --- | --- | --- |
|  |  | **Change from baseline** | **Difference from placebo** | | | **Change from baseline** | **Difference from placebo** | | |
|  |  | **Mean (SE)** | **Mean (SE)** | **95% CI** | ***P* value** | **Mean (SE)** | **Mean (SE)** | **95% CI** | ***P* value** |
| 1 | Placebo (n=42) | -2.1 (0.59) | — | — | — | -0.1 (0.10) | — | — | — |
|  | Fezolinetant |  |  |  |  |  |  |  |  |
|  | 15 mg BID (n=45) | -3.8 (0.56) | -1.7 (0.78) | -3.23, -0.17 | .0297 | -0.4 (0.09) | -0.3 (0.13) | -0.60, -0.08 | .0098 |
|  | 30 mg BID (n=43) | -5.3 (0.58) | -3.1 (0.78) | -4.66, -1.58 | <.0001 | -0.5 (0.10) | -0.4 (0.13) | -0.63, -0.11 | .0048 |
|  | 60 mg BID (n=43) | -5.7 (0.57) | -3.6 (0.78) | -5.10, -2.02 | <.0001 | -0.6 (0.10) | -0.5 (0.13) | -0.80, -0.28 | <.0001 |
|  | 90 mg BID (n=41) | -6.4 (0.60) | -4.2 (0.79) | -5.78, -2.66 | <.0001 | -0.8 (0.10) | -0.7 (0.13) | -1.00, -0.47 | <.0001 |
|  | 30 mg QD (n=41) | -3.3 (0.60) | -1.2 (0.79) | -2.76, 0.36 | .1378 | -0.1 (0.10) | -0.1 (0.13) | -0.32, 0.21 | .6761 |
|  | 60 mg QD (n=44) | -4.7 (0.56) | -2.6 (0.78) | -4.13, -1.05 | .0010 | -0.4 (0.09) | -0.3 (0.13) | -0.57, -0.05 | .0188 |
|  | 120 mg QD (n=44) | -4.7 (0.58) | -2.6 (0.78) | -4.10, -1.04 | .0011 | -0.4 (0.10) | -0.4 (0.13) | -0.61, -0.10 | .0067 |
| 2 | Placebo (n=42) | -3.7 (0.60) | — | — | — | -0.2 (0.12) | — | — | — |
|  | Fezolinetant |  |  |  |  |  |  |  |  |
|  | 15 mg BID (n=44) | -5.3 (0.57) | -1.6 (0.79) | -3.20, -0.07 | .0405 | -0.5 (0.12) | -0.4 (0.17) | -0.68, -0.03 | .0335 |
|  | 30 mg BID (n=42) | -6.8 (0.59) | -3.1 (0.80) | -4.70, -1.55 | .0001 | -0.7 (0.12) | -0.6 (0.17) | -0.91, -0.25 | .0007 |
|  | 60 mg BID (n=43) | -6.8 (0.58) | -3.1 (0.80) | -4.66, -1.52 | .0001 | -1.0 (0.12) | -0.8 (0.17) | -1.14, -0.48 | <.0001 |
|  | 90 mg BID (n=39) | -7.3 (0.61) | -3.7 (0.81) | -5.26, -2.07 | <.0001 | -1.2 (0.13) | -1.1 (0.17) | -1.41, -0.74 | <.0001 |
|  | 30 mg QD (n=41) | -5.4 (0.61) | -1.7 (0.81) | -3.34, -0.15 | .0325 | -0.3 (0.13) | -0.2 (0.17) | -0.50, 0.17 | .3227 |
|  | 60 mg QD (n=43) | -6.0 (0.57) | -2.4 (0.80) | -3.92, -0.78 | .0035 | -0.6 (0.12) | -0.4 (0.17) | -0.77, -0.11 | .0085 |
|  | 120 mg QD (n=43) | -5.7 (0.59) | -2.1 (0.79) | -3.61, -0.49 | .0102 | -0.8 (0.12) | -0.6 (0.17) | -0.95, -0.30 | .0002 |

^a^ From mixed effect model for repeated measures, with change from baseline as the dependent variable and treatment group, visit, and smoking status as factors and baseline measurement as a covariate, as well as interaction of treatment by week and an interaction of baseline measurement by week.

**Table S2.** Secondary Efficacy Outcomes: Frequency of Mild/Moderate/Severe VMS and Severity per 24 Hours, Full Analysis Set

| **Wk** | **Treatment group (n)** | **Frequency of mild/moderate/severe VMS per 24 hours^a^** | | | | **Severity of mild/moderate/severe VMS per 24 hours^a^** | | | |
| --- | --- | --- | --- | --- | --- | --- | --- | --- | --- |
|  |  | **Change from baseline** | **Difference from placebo** | | | **Change from baseline** | **Difference from placebo** | | |
|  |  | **Mean (SE)** | **Mean (SE)** | **95% CI** | ***P* value** | **Mean (SE)** | **Mean (SE)** | **95% CI** | ***P* value** |
| 4 | Placebo (n=42) | -4.0 (0.63) | — | — | — | -0.3 (0.13) | — | — | — |
|  | Fezolinetant |  |  |  |  |  |  |  |  |
|  | 15 mg BID (n=40) | -5.7 (0.60) | -1.7 (0.84) | -3.36, -0.06 | .0428 | -0.8 (0.12) | -0.5 (0.18) | -0.85, -0.16 | .0046 |
|  | 30 mg BID (n=41) | -7.0 (0.62) | -3.0 (0.84) | -4.65, -1.34 | .0004 | -1.0 (0.13) | -0.6 (0.18) | -0.99, -0.29 | .0003 |
|  | 60 mg BID (n=40) | -7.2 (0.61) | -3.2 (0.84) | -4.88, -1.58 | .0001 | -1.1 (0.13) | -0.8 (0.18) | -1.14, -0.45 | <.0001 |
|  | 90 mg BID (n=37) | -8.1 (0.64) | -4.1 (0.85) | -5.73, -2.37 | <.0001 | -1.4 (0.13) | -1.1 (0.18) | -1.41, -0.70 | <.0001 |
|  | 30 mg QD (n=40) | -6.0 (0.64) | -2.0 (0.85) | -3.64, -0.28 | .0225 | -0.8 (0.13) | -0.4 (0.18) | -0.77, -0.06 | .0209 |
|  | 60 mg QD (n=43) | -6.8 (0.60) | -2.8 (0.84) | -4.49, -1.19 | .0008 | -0.9 (0.12) | -0.6 (0.18) | -0.93, -0.24 | .0011 |
|  | 120 mg QD (n=42) | -7.1 (0.62) | -3.1 (0.84) | -4.70, -1.42 | .0003 | -1.1 (0.13) | -0.7 (0.18) | -1.07, -0.38 | <.0001 |
| 12 | Placebo (n=37) | -5.7 (0.58) | — | — | — | -0.8 (0.15) | — | — | — |
|  | Fezolinetant |  |  |  |  |  |  |  |  |
|  | 15 mg BID (n=38) | -7.1 (0.54) | -1.4 (0.76) | -2.90, 0.09 | .0653 | -1.2 (0.15) | -0.4 (0.21) | -0.80, 0.02 | .0596 |
|  | 30 mg BID (n=37) | -7.6 (0.57) | -2.0 (0.76) | -3.46, -0.47 | .0102 | -1.3 (0.15) | -0.4 (0.21) | -0.85, -0.03 | .0360 |
|  | 60 mg BID (n=31) | -8.5 (0.57) | -2.9 (0.77) | -4.38, -1.36 | .0002 | -1.6 (0.15) | -0.7 (0.21) | -1.15, -0.32 | .0006 |
|  | 90 mg BID (n=31) | -8.8 (0.59) | -3.2 (0.78) | -4.72, -1.67 | <.0001 | -1.6 (0.16) | -0.8 (0.21) | -1.23, -0.39 | .0002 |
|  | 30 mg QD (n=33) | -7.4 (0.59) | -1.8 (0.78) | -3.30, -0.25 | .0231 | -1.0 (0.15) | -0.2 (0.21) | -0.62, 0.21 | .3227 |
|  | 60 mg QD (n=36) | -8.0 (0.55) | -2.4 (0.76) | -3.88, -0.87 | .0021 | -1.3 (0.15) | -0.5 (0.21) | -0.89, -0.07 | .0220 |
|  | 120 mg QD (n=36) | -8.1 (0.57) | -2.5 (0.76) | -3.96, -0.97 | .0013 | -1.3 (0.15) | -0.4 (0.21) | -0.84, -0.02 | .0380 |

^a^From mixed effect model for repeated measures with change from baseline as the dependent variable and treatment group, visit, smoking status as factors and baseline measurement as a covariate, as well as interaction of treatment by week and an interaction of baseline measurement by week.

**Table S3.** Liver Function Tests, Safety Analysis Set

| **Parameter** | **Placebo (n=42)** | **Fezolinetant, n (%)** | | | | | | |
| --- | --- | --- | --- | --- | --- | --- | --- | --- |
|  |  | **15 mg BID (n=43)** | **30 mg BID (n=41)** | **60 mg BID (n=41)** | **90 mg BID (n=40)** | **30 mg QD (n=41)** | **60 mg QD (n=43)** | **120 mg QD (n=42)** |
| AST or ALT, n (%) |  |  |  |  |  |  |  |  |
| >3 × ULN | 0 | 0 | 1 (2.4) | 3 (7.3) | 2 (5.0) | 0 | 1 (2.3) | 2 (4.8) |
| >5 × ULN | 0 | 0 | 0 | 1 (2.4) | 2 (5.0) | 0 | 1 (2.3) | 1 (2.4) |
| >8 × ULN | 0 | 0 | 0 | 1 (2.4) | 1 (2.5) | 0 | 1 (2.3) | 0 |
| >10 × ULN | 0 | 0 | 0 | 0 | 0 | 0 | 1 (2.3) | 0 |
| Total bilirubin, n (%) |  |  |  |  |  |  |  |  |
| >1.5 × ULN | 0 | 0 | 1 (2.4) | 0 | 0 | 0 | 1 (2.3) | 0 |
| >2.0 × ULN | 0 | 0 | 0 | 0 | 0 | 0 | 0 | 0 |
| Alkaline phosphatase >1.5 × ULN, n (%) | 0 | 0 | 2 (4.9) | 1 (2.4) | 0 | 2 (4.9) | 2 (4.7) | 0 |
| ALT and/or AST >3 × ULN *and* total bilirubin 2 × ULN, n (%) | 0 | 0 | 0 | 0 | 0 | 0 | 0 | 0 |
| ALT and/or AST >3 × ULN *and* ALP <2 × ULN *and* total bilirubin >2 × ULN, n (%) | 0 | 0 | 0 | 0 | 0 | 0 | 0 | 0 |

ALT=alanine aminotransferase; AST=aspartate aminotransferase; ULN=upper limit of normal.

**Figure S1.** Response Rates and Odds of Response with Fezolinetant Versus Placebo Based on ≥50% Reduction in Frequency of Moderate/Severe VMS at Last On-Treatment Visit, Full Analysis Set

**
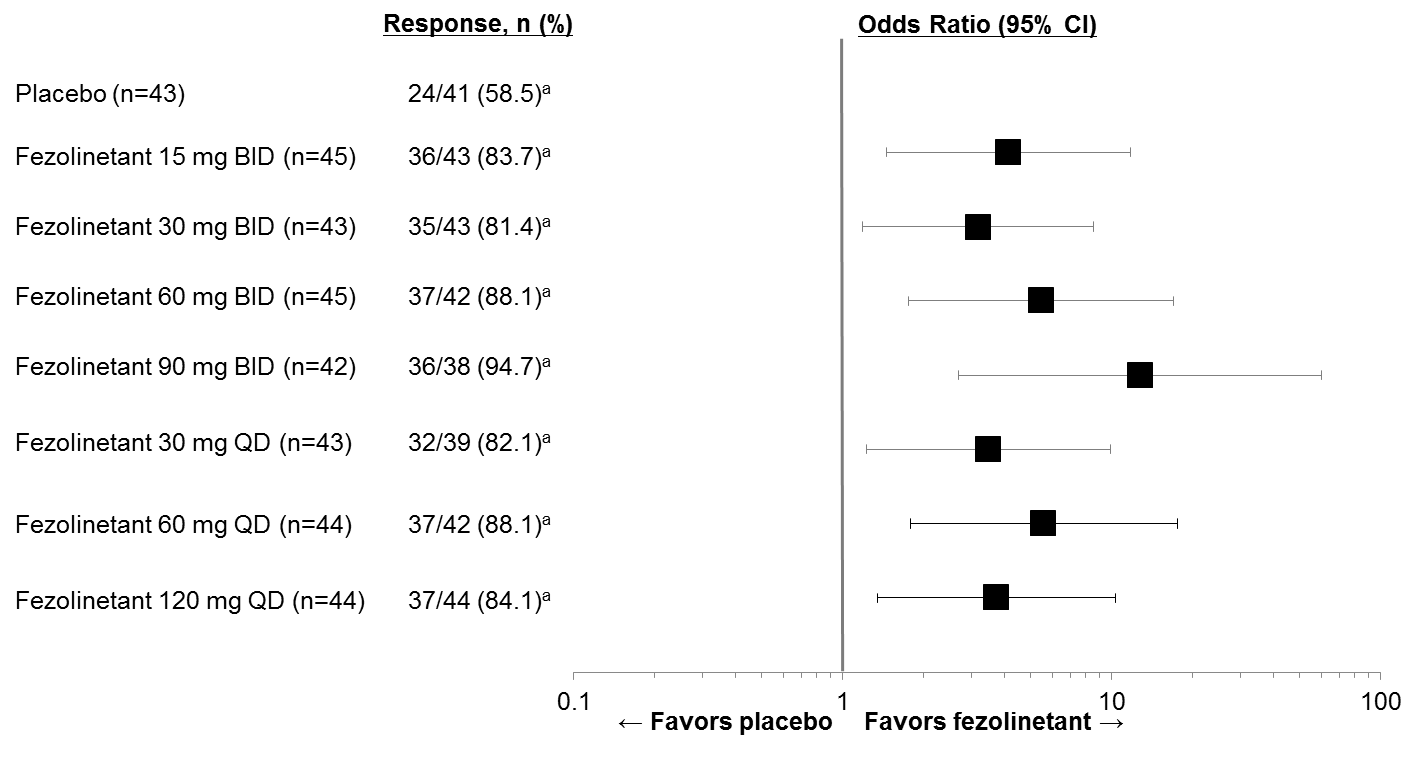
**

VMS=vasomotor symptoms.

^a^*P*<.05 for all pairwise comparisons of fezolinetant vs placebo, with no adjustments for multiplicity.

**Figure S2.** Endometrial Thickness, Safety Analysis Set


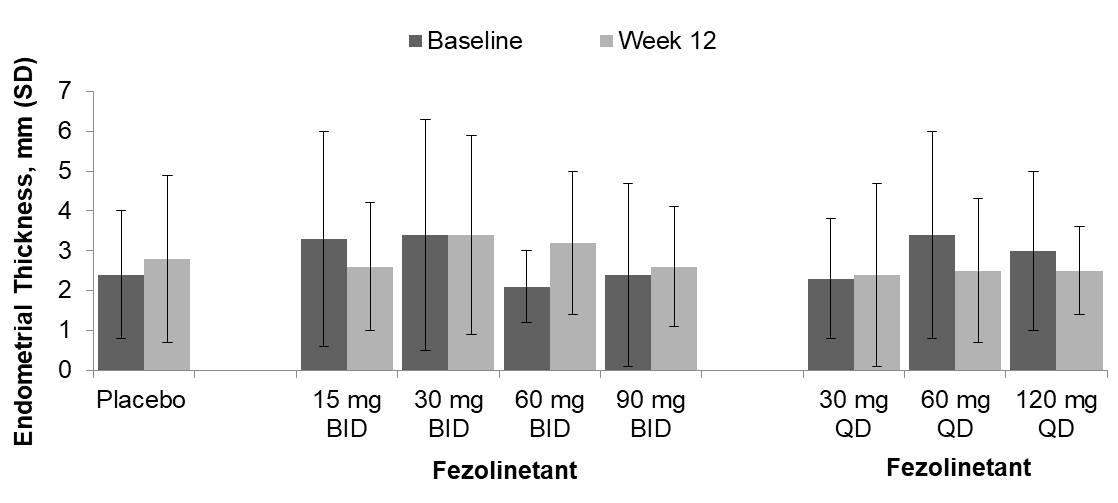

Supplement: Supplemental Digital Content [file menop-27-382-s003.docx]
